# Transition metals induce control of enhanced NLO properties of functionalized organometallic complexes under laser modulations

**DOI:** 10.1038/s41598-020-71769-2

**Published:** 2020-09-17

**Authors:** S. Taboukhat, N. Kichou, J.-L. Fillaut, O. Alévêque, K. Waszkowska, A. Zawadzka, A. El-Ghayoury, A. Migalska-Zalas, B. Sahraoui

**Affiliations:** 1 MOLTECH-Anjou, UMR 6200, CNRS, Univ Angers, 2 Bd Lavoisier, 49045 Angers, France; 2 Univ Rennes, CNRS, ISCR-UMR 6226, 35000 Rennes, France; 3 Department of Chemistry, Faculty of Sciences, University of Mouloud Mammeri, 15000 Tizi-ouzou, Algeria; 4 Institute of Physics, Faculty of Physics, Astronomy and Informatics, Nicolaus Copernicus University, 5 Grudziadzka, 87-100 Torun, Poland; 5 Faculty of Science and Technology, Jan Dlugosz University in Czestochowa, Al. Armii Krajowej 13/15, 42-200 Czestochowa, Poland

**Keywords:** Materials science, Optics and photonics, Physics

## Abstract

The molecular engineering of organometallic complexes has recently attracted renewed interest on account of their potential technological applications for optoelectronics in general and optical data storage. The transition metal which induces control of enhanced nonlinear optical properties of functionalized organometallic complexes versus not only the intensity but also the polarization of the incident laser beam is original and important for all optical switching. This makes organometallic complexes valuable and suitable candidates for nonlinear optical applications. In the present work, we report the synthesis and full characterization of four organometallic complexes consisting of *N, N*-dibutylamine and azobenzene fragments but differ by auxiliary alkynyl ligands or metal cations. Thus, a ferrocenyl derivative **1** and three ruthenium complexes **2–4** have been prepared. The nonlinear optical properties of the four new azo-based ruthenium and iron organometallic complexes in the solid state, using polymethylmethacrylate as matrix, have been thoroughly studied. This concept is extended to computing the HOMO and LUMO energy levels of the considered complexes, dipole moment, first and second order hyperpolarizabilities using the 6–31 + G(d,p) + LANL2DZ mixed basis set. The second and third nonlinear optical properties of the resulting polymer composites were obtained by measuring SHG and THG response by means of the Maker fringe technique using a laser generating at 1,064 nm with a 30 ps pulse duration. The values of the second and third order NLO susceptibilities of the four organometallic complexes were found to be higher than the common references used. Theoretical calculation shows that the large first and second order hyperpolarizablities are caused by strong intramolecular charge transfer between the transition metal parts and the ligands though a conjugated transmitter. These results indicate that the present organometallic complexes are valuable candidates for optoelectronic and photonic applications.

## Introduction

The organometallic complexes are among the most studied materials in nonlinear optics (NLO)^1,2^. Their optoelectronic properties are highly dependent on the metal-d orbital occupation and energy levels, which can be easily modulated through the choice of the metal, its oxidation state and the surrounding ligands^3^. Among these complexes, the family of alkynyl-metal complexes that can be defined as carbon-rich transition metal complexes which have a nearly linear M–C≡C–R structure demonstrated particularly suitable characteristics for NLO studies due to an efficient electronic coupling between the metal and distant groups via the π-conjugate pathway. This electronic coupling, known as the metal-to-ligand charge transfer (MLCT), is highly dependent on the degree of overlap between the filled metal d orbitals and the unoccupied π -orbitals of the alkynyl moiety^4–6^. The physical and optoelectronic properties of the metal alkynyl complexes can be optimized through a careful design, related to the choice of both the organic and the metallic end groups, for example by using iron or ruthenium, and of the organic bridges of the π-conjugated entity^7–12^. This diversity could be used for different applications such as optoelectronics, quantum electronics, and optical communications and in the field of molecular engineering of functional materials^13–15^. Moreover it could be also useful for tuning the properties of organometallic based magnetics for spintronic and quantum computing devices^16^ and also electronic and optical properties for photovoltaic and optoelectronic applications^17^.

Much research has been carried out on the study of azobenzene derivatives which, thanks to their reversible and stable *trans–cis* photoisomerisation over many cycles, are among the most studied photochromic compounds in recent years. Azobenzene systems possess also obvious advantages in the field of NLO, due to their high intramolecular charge transfer and their high hyperpolarizabilities^18–20^ which can be coupled with the photoinduced trans–cis isomerization. Hence, they could function as NLO molecular switches by applying light of different wavelengths to obtain varying amounts of cis and trans isomers. On the other hand, donor–acceptor substituted azo dyes, which are molecules with easily polarizable electrons, show large second-order nonlinearities^21,22^. A major advantage is also the possibility of inducing a noncentrosymmetric structure within the material, for example by orientation by the Corona poling technique, because obtaining macroscopic quadratic NLO properties requires breaking the centrosymmetry of the medium by orientation of the chromophores. These properties of azobenzene derivatives give the possibilities of many applications in optical switching and data storage^23,24^.

We report herein the synthesis and full characterization of four new azo-based ruthenium and iron organometallic complexes. The nonlinear optical (NLO) responses of these complexes, related to metal acetylide complexes, have been evaluated by means of second and third harmonic generations (SHG, THG) experimental techniques and also quantum chemical calculations. We undertook this study to better understand the physical contribution of the metal center and its coordination sphere to the NLO susceptibilities of the complexes. The parameters responsible for second and third-order nonlinear-optical effects such as second and third harmonic generation are the first and second order hyperpolarizabilities. A conventional key to achieving high values of these parameters in metal acetylide complexes is to lengthen the π-conjugate system and strengthen the donor–acceptor character^21, 25–30^. In this work the effect of the metal transition complexation on the intramolecular charge transfer as well as the role of auxiliary ligands have been investigated. The relations between theoretically calculated NLO properties and the experimental obtained second and third order susceptibilities are presented. Both results indicate that these molecular materials are valuable candidates for application in optoelectronics and photonics^31^.

## Experimental studies

All new compounds are air stable in the solid state and reasonably air stable in solution, but the vinylidene intermediates are not air stable and all reactions were performed under an argon atmosphere. All the solvents were of HPLC grade, further purified in a solvent system containing drying columns or dried over 4 Å molecular sieves. All commercially available reagents were used without further purification. The starting materials, ferrocenylacetylene^32^, cis-[Ru(dppm)_2_Cl_2_]^33^, *cis*-[RuCl_2_(PPh_3_)_2_(4,4′-dimethyl-2,2′-bipyridine)]^34^ and RuCl(dppe) *η*^*5*^-Cp^35^, (E)-N,N-dibutyl-4-((4-bromophenyl)diazenyl)aniline **5**^36^ and (E)-N,N-dibutyl-4-((4-ethynylphenyl)diazenyl)aniline^21,37^, **6** were prepared by literature methods.

Flash column chromatography was performed on silica gel (high-purity grade, 230–400 mesh, 40–63 μm, Sigma-Aldrich) according to a standard technique. Nuclear magnetic resonance spectra (^1^H, ^13^C and ^31^P) were recorded on a Bruker spectrometer (400 MHz). Chemical shifts for ^1^H and ^13^C spectra are recorded in parts per million and are calibrated to solvent residual peaks (for example: CHCl_3_: ^1^H 7.26 ppm, ^13^C 77.16 ppm) according to H. E. Gottlieb, V. Kotlyar and A. Nudelman^38^. Multiplicities are indicated by s (singlet), bs (broad singlet), d (doublet), t (triplet), q (quadruplet), quint (quintuplet) and m (multiplet). Coupling constants, J, are reported in Hertz. Exact mass was obtained through Matrix Assisted Laser Desorption Ionization Time Of Flight Mass Spectrometry (MALDI-TOF MS).

### Syntheses

Synthesis of **1**: 250 mg (1.2 mmol) of ferrocenylacetylene^32^ and 466 mg (1.2 mmol) of **5**^36^ were dissolved in 20 mL of THF and 20 mL of Et_3_N in a round bottom flask. This flask was purged three times with argon. 11 mg (60 μmol) of copper iodide and 41 mg (60 μmol) of bis(triphenylphosphine)palladium(II) dichloride were added and the reaction mixture was stirred overnight at 50 °C. After the solvent was removed, the residue was extracted 3 times with 25 mL of CH_2_Cl_2_ and washed with 50 ml of water, and then dried over Na_2_SO_4_. The resulting mixture was concentrated under reduced pressure. The crude product was purified by chromatography on silica gel eluted with ether/petroleum ether (1/4, v/v) to give **1**. Red–orange crystalline powder, yield 366 mg, 59%. ^1^H NMR (400 MHz, CDCl_3_) δ 7.89 (d, 7.8 Hz, 2H), 7.84 (d, 7.8 Hz, 2H), 7.62 (d, 8.0 Hz, 2H), 6.72, (d, 8.0 Hz, 2H), 4.56 (s, 2H), 4.29 (br. s., 7H), 3.39 (t, 7.8 Hz, 4H), 1.66 (m, 4H), 1.43 (m, 4H), 1.01 (t, 7.7 Hz, 6H). ^13^C NMR (101 MHz, CDCl_3_) δ 152.22, 150.69, 143.21, 132.04, 125.37, 124.59, 122.19, 111.13, 90.25, 86.12, 71.49, 70.03, 68.96, 65.25, 50.99, 29.53, 20.34, 14.01. MALDI-TOF MS calculated for C_32_H_35_FeN_3_ *m*/*z*: 517.22, found: 517.56.

Preparation of **2**: cis-[Ru(dppm)_2_Cl_2_]^33^, (300 mg, 0.22 mmol) was added to **6**^21,37^ (74 mg, 0.22 mmol) and KPF_6_ (0.6 mmol) in CH_2_Cl_2_ (25 mL) and stirred for 16 h. The dark red solution was filtered and the solvent removed in vacuo. The solid residue was washed with deoxygenated diethylether (3 × 10 mL) to remove any excess of **6** and then redissolved in CH_2_Cl_2_, and K_2_CO_3_ (0.6 mmol) was added to the vinylidene solution (^31^P{^1^H}NMR: δ (ppm) -16.7 (s, PPh_2_)) and the stirring continued for a further 2 h. The resulting red–orange solution was filtered before 20 ml of heptane was added. The solid thus obtained was isolated by filtration, washed with pentane, and dried under vacuum to give the product as a red–orange crystalline powder. Complex **2**, yield 163 mg, 60%. ^31^P NMR (162 MHz, CDCl_3_) δ − 6.7 ppm. ^1^H NMR (400 MHz, CDCl_3_) δ 7.81 (d, 7.8 Hz, 2H), 7.53–7.44 (m, 16H) 7.32 (t, 8.0 Hz, 4H), 7.30 (t, 8.0 Hz, 4H), 7.28 (t, 8.0 Hz, 8H), 7.21(t, 8.0 Hz, 8H), 7.11 (t, 7.2 Hz, 2H), 6.72 (d, 7.8 Hz, 2H), 6.14 (d, 7.8 Hz, 2H), 4.96 (m., 4H), 3.38 (d, 7.8 Hz, 2H), 1.66 (m., 4H) 1.42 (m., 4H), 1.02 (t., 7.0 Hz, 6H). ^13^C NMR (101 MHz, CDCl_3_) δ 149.8, 148.6, 143.5, 134.2 (quint, J_CP_ = 11 Hz), 133.8 (quint, J_CP_ = 11 Hz), 133.4, 132.3, 129.28, 127.81, 127.56, 124.54, 121.22, 114.59, 111.13, 50.97, 50.32 (t., J_CP_ = 10 Hz), 29.55, 20.36, 14.02. Anal. Calcd for C_72_H_70_ClN_3_P_4_Ru: C, 69.87; H, 5.70; N, 3.39. Found: C, 69.05; H, 5.93, N, 3.14.

Preparation of **3**: A deoxygenated solution of the complex *cis*-[RuCl_2_(PPh_3_)_2_(4,4′-dimethyl-2,2′-bipyridine)]^34^ (200 mg, 0.24 mmol), **6** (135 mg, 0.4 mmol) and KPF_6_ (43.1 mg, 0.23 mmol) in 20 mL of CH_2_Cl_2_/MeOH (1/1) was stirred for 48 h at room temperature. The mixture was filtered off in celite column, concentrated to ca. 1 mL and precipitated with heptane. The solid obtained was washed with pentane, dried under vacuum and dissolved in CH_2_Cl_2_ (15 mL) (^31^P{^1^H} NMR (162 MHz, CDCl_3_): 20.9 ppm (singlet, PPh_3_), − 143.92 (septet, PF_6_, ^1^*J*_PF_ = 710 Hz). K_2_CO_3_ (2 mmol) was added and the reaction mixture was stirred for another 6 h. The crude product was purified by chromatography on silica gel eluted with CH_2_Cl_2_/petroleum ether (1/2, v/v) to give a red–orange solid identified as **3**. Yield 130 mg, 45%. ^31^P NMR (162 MHz, CDCl_3_) δ, 29.5 ppm. ^1^H NMR (400 MHz, CDCl_3_) δ 8.90 (d, 5.5 Hz, 1H), 8.22 (d, 5.5 Hz, 1H), 7.81 (d, 7.8 Hz, 2H), 7.72–7.46 (unresolved m., 20H), 7.16 – 6.93 (unresolved m.,16H), 6.72 (d, 7.8 Hz, 2H), 6.63 (d, 5.5 Hz, 1H), 5.95 (d, 5.5 Hz, 1H), 3.38 (t, 7.2 Hz, 4H), 2.33 (s, 3H), 2.21 (s, 3H), 1.65 (s, 4H), 1.42 (s, 4H), 1.00 (s, 7.8 Hz, 6H).^13^C NMR (101 MHz, CDCl_3_) δ 156.59, 155.53, 154.88, 151.93, 149.72, 144.73, 143.51, 142.60, 134.18 (t, 4.2 Hz), 133.95, 133.03, 132.14, 132.05, 131.97, 131.94, 128.57, 128.45, 128.04, 127.05 (t, 4.0 Hz), 124.55, 120.63, 120.37, 111.14, 50.95, 29.56, 21.03, 20.65, 20.35, 14.02. Anal. Calcd for C_70_H_68_ClN_5_P_2_Ru: C, 71.38; H, 5.82; N, 5.95. Found: C, 71. 96; H, 5.58; N, 5.74.

Preparation of **4**: A solution of RuCl(dppe)*η*^*5*^-Cp^35^, (380 mg, 0.5 mmol), **6** (200 mg, 0.6 mmol), and KPF_6_ (2.5 mmol) in 15 mL of CH_2_Cl_2_ was stirred for 48 h at room temperature under argon. Deoxygenated diethyl ether (2 × 50 mL) was added and the mixture filtered. The filtrate was dissolved in CH_2_Cl_2_ (15 mL) ^31^P{^1^H} NMR (singlet at 42 ppm (PPh_3_), − 143.87 (septet, PF_6_, ^1^*J*_PF_ = 709 Hz). Then K_2_CO_3_ (2 mmol) was added and the reaction mixture was stirred for another 6 h. The crude product was purified by chromatography on silica gel eluted with diethylether/petroleum ether (1/2, v/v) to give **4**. Red solid, yield 250 mg, 48%. ^31^P NMR (162 MHz, CDCl_3_) δ, 50.2 ppm (s, PPh_3_). ^1^H NMR (400 MHz, CDCl_3_) δ 7.83 (d, 7.8 Hz, 2H), 7.70 (d., 7.8 Hz, 2H), 7.51 (m, 10H ), 7.23 (m, 8H ), 7.16 (m, 12H), 6.71 (d, 7.8 Hz, 2H), 4.37 (s, 5H) 3.38 (t, 7.0 Hz, 4H), 1.66 (m, 4H), 1.42 (m, 4H), 1.01 (t, 7.1 Hz, 6H). ^13^C NMR (101 MHz, CDCl_3_) δ 149.88, 149.19, 143.47, 138.77 (m, ^1^*J*_CP_ + ^*3*^*J*_CP_ = 42 Hz), 133.82, 130.93, 128.81, 128.47, 127.27, 124.66, 122.57, 122.02, 116.33, 111.13, 85.32, 50.96, 29.51, 20.35, 14.01. Anal. Calcd for C_63_H_61_N_3_P_4_Ru: C, 71.78; H, 5.83; N, 3.99. Found: C, 71. 96; H, 5.58; N, 3.74.

### Electrochemical experiments

Electrochemical experiments were carried out with a Biologic SP-150 potentiostat driven by the EC-Lab software including ohmic drop compensation. Cyclic Voltammetry (CV) was performed in a three-electrode cell controlled at a temperature of 293 K in a glove box containing dry, oxygen-free (< 1 ppm) argon. Working electrodes were glassy carbon planar disk electrodes (Ø = 3 mm). Counter electrodes were platinum wires. Reference electrodes were Ag/AgNO_3_ (0.01 M CH_3_CN). Experiments were recorded in dry HPLC-grade acetonitrile with tetrabutylammonium hexafluorophosphate (Bu_4_NPF_6_, electrochemical grade, Fluka) as supporting electrolyte. All the potential reported were calibrated versus ferricinium/ferrocene couple (Fc^+^/Fc) (IUPAC Recommendation)^39^. Based on repetitive measurements, absolute errors on potentials were found to be around ± 5 mV.

### Preparation of host–guest films of complexes and optical absorption measurements

For the host–guest films preparation, the various chromophores and PMMA (Sigma-Aldrich, Mw = 15,000 g/mol) have been dissolved in dichloromethane at concentration of 50 g/L. The concentration of the compounds was 100 µmol towards 1 g of PMMA. The solutions were deposited using the spin-coater (SCS G3) at 1,000 rpm on BK 7 glass plates substrates with 1 mm thickness (which were cleaned in distilled water using ultrasonic bath, acetone, and ethanol) and then were dried. Obtained guest–host polymer films were kept at room temperature during two days in order to eliminate any remaining of solvent. The thickness of deposited films was calculated by the profilometer (Dektak 6M, Veeco).

We used Lambda 950 UV/Vis/NIR spectrophotometer (PerkinElmer) with the range 300–1,200 nm in order to measure the absorption spectra of the samples.

## NLO studies

### SHG and THG measurements

After the preparation of the thin films, we used the corona poling technique in order to orientate the chromophores at the thin layer surface. First, they were heated on a hot plate at selected poling temperature of 100 °C. Then an external electric field was provided by applying a voltage of + 5 kV to a tungsten needle fixed at 1 cm above the polymer surface, while the electrode under the glass substrate was grounded. With the remaining electric field, the heater was switched off, and the sample was cooled down to room temperature, the corona field was turned off.

In order to evaluate the nonlinear optical response of our metal complexes **1**, **2**, **3** and **4**, we use the Third and Second Harmonic Generation (SHG & THG), which are based on the Maker Fringe technique^40^. In this Harmonic Generation technique, an incident laser beam at the frequency $\upomega$ interacts with a nonlinear medium in order to generate another beam with double and triple frequencies.

**Figure 1:**
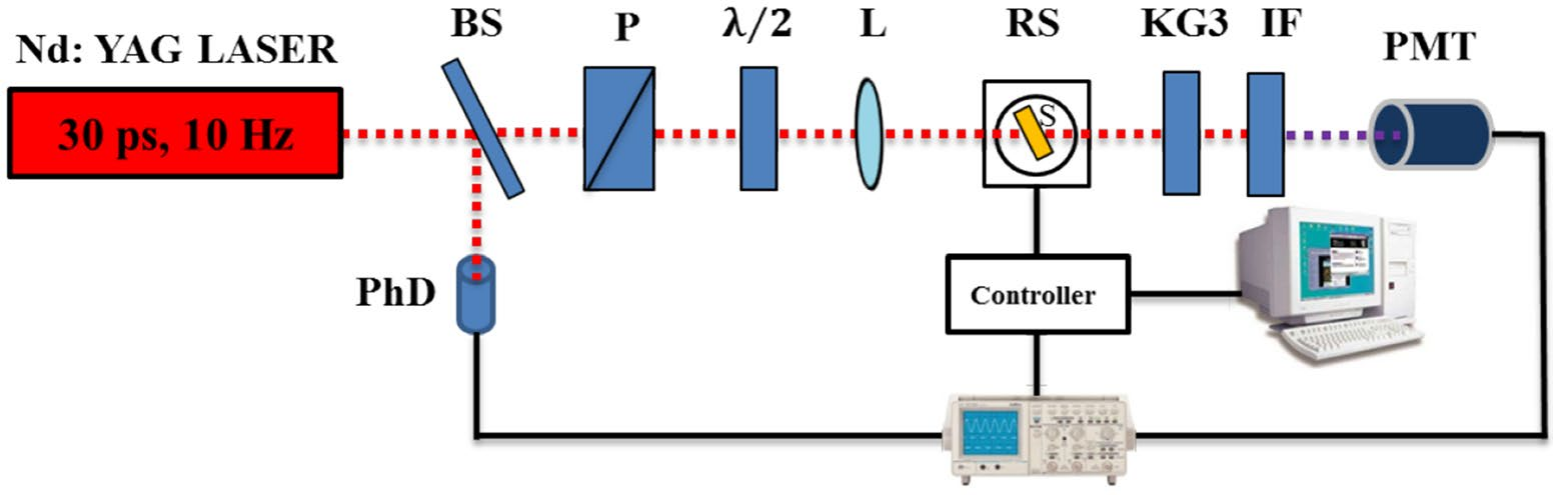
Experimental setup for the THG and SHG measurements: (BS) the beam splitters, (P) the Glan polarizers, (PhD) the photodiode, ($\uplambda /2$) the half wave plate, (L) the lens, (S) the sample, (RS) the rotation stage, (F) the neutral density filters, (KG3) the KG3 filter, (IF) the interference filter, (PMT) the photomultiplier tube.

We used a Nd:YAG laser working at 1,064 nm with 30 ps pulse duration and we employed 10 Hz repetition rate (Fig. 1). The input energy of laser pulses was controlled by laser power/energy meter (LabMax TOP, COHERENT) to be 95 μJ for SHG and 140 μJ for THG measurements respectively. The thin films were mounted on a rotating stage and the beam was focalized on them. A fast photodiode measured a portion of the input beam in order to synchronize the rotation and the fundamental beam. The interference filter at 532 nm (or 355 nm) was used to select the desired wavelength of light^20,41–44^ and was used to cut the pump beam before the photomultiplier, that allowed the third harmonic or the second harmonic generated signal to be detected. The polarization of second and third harmonic was controlled by polarizer placed before the photomultiplier. We used established reference materials, quartz glass plate and silica glass plate respectively, for the second harmonic generation and third harmonic generation measurements^45^.

### Theoretical quantum chemical calculations of NLO properties

For nonlinear optical properties calculation of the first and second order hyperpolarizabilities (β, γ), the time-dependent Hartree–Fock (TDHF) level of theory with functional LanL2DZ for transition metals Ru Fe and 6–31 + G(d,p) functional for the other concerned atoms (C, H, N, P, Cl) have been used. Indeed, in order to obtain good theoretical results we combined two methods (LANL2DZ functional^46^ for transition metals and all-electron basis sets for all other non-transition-metal atoms), which is prevalent in computations for transition-metal-containing complexes. In order to get a precise estimation of the hyperpolarizabilities one should take into account d polarization functions on the carbon and nitrogen atoms and the addition of p functions on hydrogen atoms and diffuse functions. Calculations were performed for structures optimized by density functional theory DFT/B3LYP at the 6–31 + G(d,p) + LANL2DZ mixed basis set. The hybrid-GGA functional B3LYP^47^ gives the best performance in predicting the good structure and HOMO, LUMO properties. All calculations were done using GAUSSIAN 09 program package.

## Results and discussion

### Synthesis and characterization

**Figure 2:**
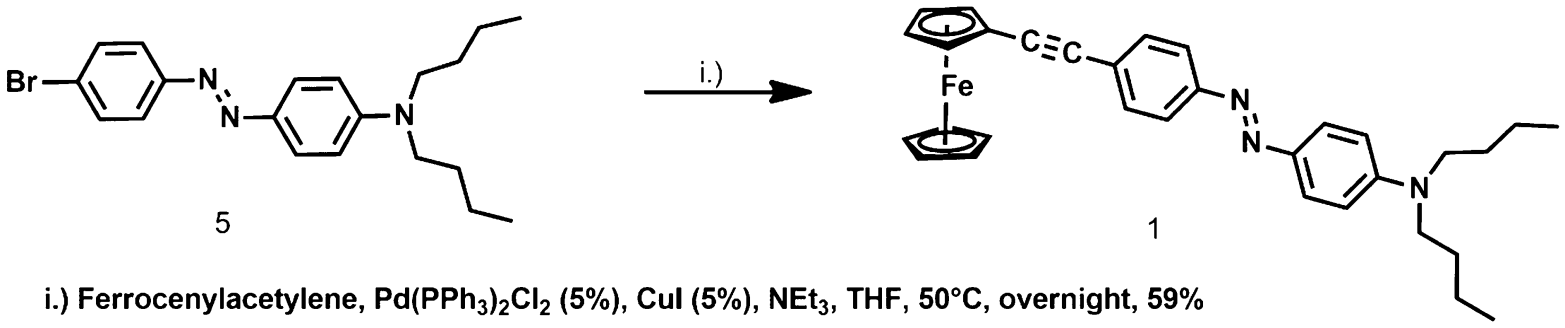
Synthesis of the ferrocenyl derivative **1**.

The catalytic coupling of ferrocenylacetylene^32^ with (E)-N,N-dibutyl-4-((4-bromophenyl)diazenyl)aniline **5**^36^ in NEt_3_ at 50 °C by PdCl_2_(PPh_3_)_2_/CuI catalysts gave the red–orange crystalline ferrocenyl derivative **1** (59%; Fig. 2 ). The acetylenic moiety was characterized by ^13^C{^1^H} NMR (δ 90.2 ppm (~ C_6_H_4_-C⋮C-) and 86.1 (C⋮C-Cp)).

**Figure 3:**
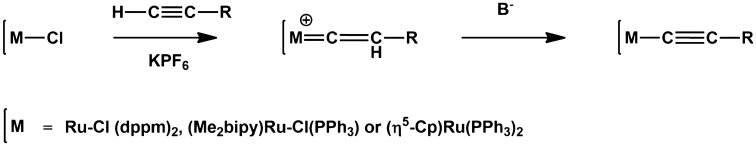
General synthetic scheme of ruthenium acetylenes derivatives.

The synthesis of the ruthenium acetylide derivatives **2–4** was attempted by the activation of the azo-containing alkyne **6** (E)-*N,N*-dibutyl-4-((4-ethynylphenyl)diazenyl)aniline^21,37^, in a two-steps procedure*, *via the formation of vinylidene intermediates (Fig. 3) followed by deprotonation, as was shown to occur for RuCl_2_(dppe)_2_ analogues^21,37^. This approach is in contrast with classical metal − carbon bond formation via a metal −halide unit and an organometallic^48^ and was preferred here because it supports easily functional groups that the alkyne bears. This route was first used to prepare the *η*^1^-ethynyl *trans*-[RuCl(-C⋮C-R)(dppm)_2_] **2**. *cis*-RuCl_2_(dppm)_2_^33^ was reacted with **6** in the presence of KPF_6_ in CH_2_Cl_2_ to give a pale green intermediate, likely to be the vinylidene [Cl(dppm)Ru = C = CH-R]^+^PF_6_^-^ salt (^31^P{^1^H}NMR: δ (ppm) -16.7 (s, PPh_2_)). On deprotonation by potassium carbonate, this intermediate led to the red ruthenium complex **2** in good yield (60%; Fig. 3) (^31^P{^1^H}NMR: δ (ppm) -6.7 (s, PPh_2_)). A singlet resonance for the Ru–C⋮C carbon nuclei (δ 114.6 ppm) confirms the presence of the alkynyl ligand.

**Figure 4:**
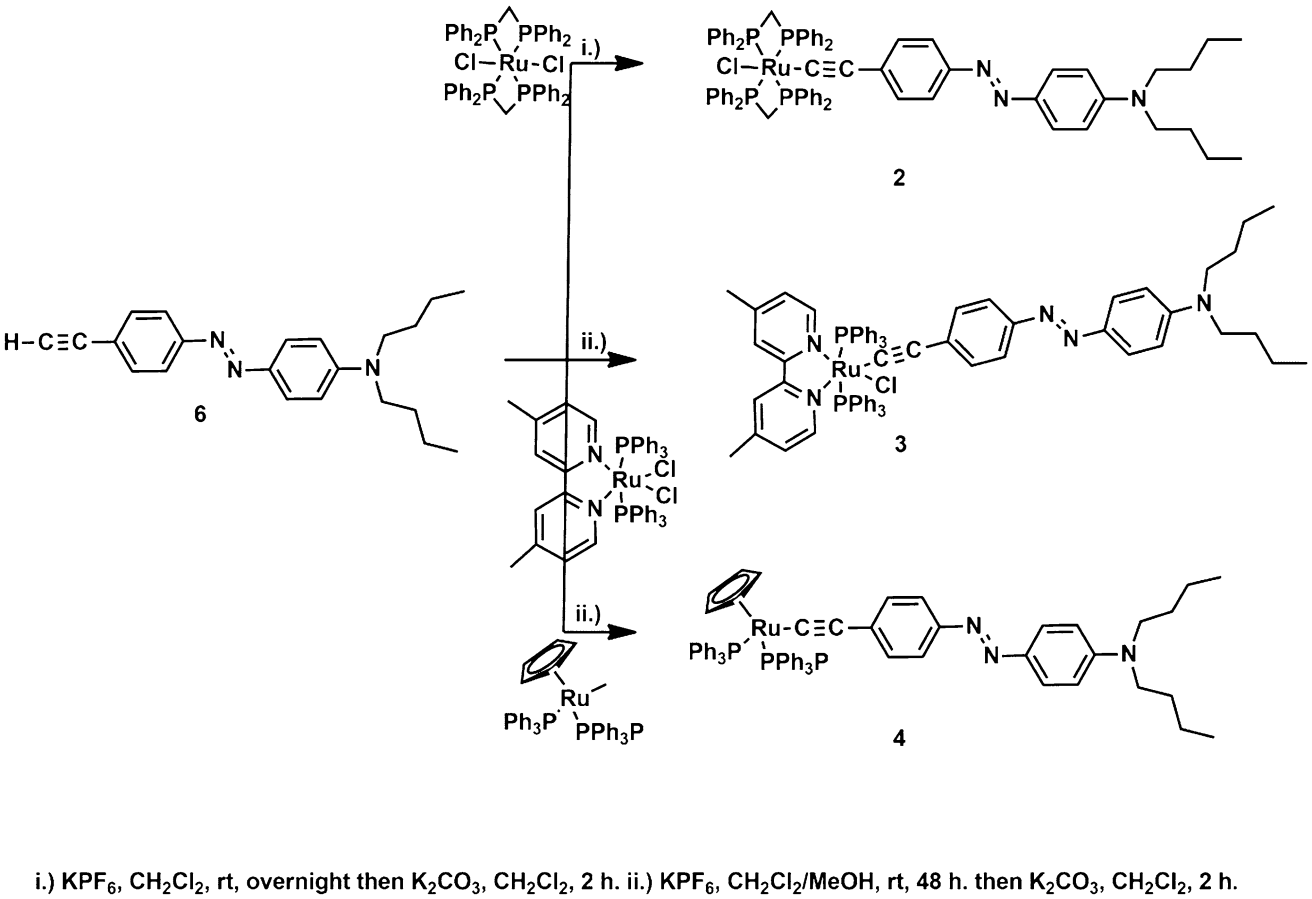
Synthesis of the ruthenium organometallic complexes **2–4**.

*cis*-[RuCl_2_(PPh_3_)_2_(4,4′-dimethyl-2,2′-bipyridine)]^34^ was used to prepare the *η*^1^-ethynyl *cis*-[RuCl(-C⋮C-R)(PPh_3_)_2_(4,4′-dimethyl-2,2′-bipyridine)], **3**. In the first step, a vinylidene complex (see Fig. 3) is obtained by chloride displacement in a CH_2_Cl_2_/MeOH 1:1, v:v solution with PF_6_^−^ salt and activation of the azo-containing alk-1-yne **6** H-C⋮C-R, at 40 °C. The formation of this vinylidene complex is confirmed by ^31^P{^1^H} NMR spectra that shows a singlet at 22.1 ppm as expected due to the equivalence of the opposite position of phosphorus atoms in a *trans* isomer. This vinylidene species decomposes slowly in air, due to reaction between the vinylidene group and atmospheric oxygen as observed for similar compounds^49^. It is quickly deprotonated upon addition of potassium carbonate in dichloromethane to give the expected *η*^1^-ethynyl derivative **3** (Fig. 4)^49^.

Finally, the reaction between RuCl(PPh_3_)_2_(*η*^*5*^-C_5_H_5_) and the alkyne **6**, in the presence of KPF_6_, has given the corresponding cationic η^1^-vinylidene complex [Ru(C = CHR)(PPh_3_)_2_(*η*^*5*^-C_5_H_5_)]^+^, the formation was attested by ^31^P{^1^H} NMR (singlet at 42 ppm). This cation is readily deprotonated to give the corresponding *η*^1^-ethynyl derivative Ru(-C⋮C-R) (PPh_3_)_2_(*η*^*5*^-C_5_H_5_), **4 (**^31^P{^1^H} NMR (singlet at 50.2 ppm).

These new alkynyl complexes were characterized by MS or satisfactory microanalyses, UV–vis spectroscopy and ^1^H-, ^31^P- and ^13^C-NMR spectroscopy (see part 1.1 and Supporting Information).

### Cyclic voltammetry

In order to gain insight into the electronic environment of the metal atom in the new alkynyl complexes, the electrochemical properties of these compounds were measured by means of cyclic voltammetry in 0.1 M tetra-butylammonium hexafluorophosphate ([n-Bu_4_N]PF_6_) methylene chloride solutions. The results are summarized in Table 1. The cyclic voltammetric scan of compound **1** exhibits one reversible oxidation wave that corresponds to the ferrocene oxidation with a half wave potential of E_1/2_ = 0.10 V (vs. FeCp_2_/FeCp_2_^+^). This value can be compared to that of free ferrocene (i.e. internal reference used for calibration), which clearly reveals an electron transfer from donor ferrocene to the ethynyl unit, which behaves as a modest electron with-drawing group: the oxidation of the iron center becomes more anodic compared to free ferrocene due to the removal of electron density. A second anodic wave is observed at E_1/2_ = 0.50 V. This wave is reversible (*I*_*p*a_/*I*_*p*c_ ≅ 1) (*I*_*p*c_ and *I*_*p*a_ are the peak cathodic and anodic currents), and is tentatively attributed to the oxidation of the azo-dialkylamino fragment.

**Table 1:** Cyclic voltammetry and linear optical data.

Complex	E1/2 [V] [ΔE, (*I*_*pc*_*/I*_*pa*_)]^a^	λmax [nm] (ε [10^3^ M^−1^ cm^−1^])
**1**	0.10 [0.70, 1]; 0.50 [0.70, 1]	461 (54)
**2**	− 0.06 [0.80, 1]; 0.27 [0.70, 1]	500 (47)
**3**	− 0.30 [0.70, 1]; 0.30 [0.70, 1]	514 (23)
**4**	− 0.05 [0.60, 1]; 0.25 [0.70, 1]; 0.50 [0.80, 1]	481 (15)

Measured in CH_2_Cl_2_. (vs. FeCp/FeCp^+^).

^a^Pt disc working, Pt wire auxiliary, Ag reference electrode; ferrocene/ferrocenium redox couple as internal reference, CH_2_Cl_2_ containing 0.1 M [n-Bu_4_N][PF_6_], 20 °C, scan rate 100 mV s^-1^ at 5.10^–4^ M.

Cyclic voltammetric scans of complexes **2** and **3** using window potentials of − 1.0 to 1.0 V each show two electrochemically reversible waves with half-wave potentials of − 0.06 V and + 0.27 V for **2**, and − 0.30 V and + 0.30 V for **3**, (vs. FeCp_2_/FeCp_2_^+^). The lowest potential oxidation waves are attributed to the one electron oxidation of the ruthenium centers. These observations are consistent with previous results^50,51^. Conversely, the highest oxidation potentials are attributed to the azo-dialkylamino fragment.

The cyclic voltammograms of the acetylide compound **4** shows a reversible oxidation wave (*I*_*p*a_/*I*_*p*c_ ≅ 1), corresponding to the Ru^II/III^ couple at a potential [E_1/2_ = − 0.05 V vs. FeCp_2_/FeCp_2_^+^] which is concordant with the data mentioned for RuX(PPh_3_)_2_(*η*^*5*^-C_5_H_5_) series^52^. A second reversible oxidation wave is observed at E_1/2_ = 0.25 V, that would correspond to the waves observed in **2** and **3**, that we attributed to the oxidation of the azo-dialkylamino fragment. Surprisingly, a third wave, quasi reversible, was observed at 0.5 V vs. FeCp_2_/FeCp_2_^+^. This wave was tentatively attributed to a further oxidation of the azo-dialkylamino fragment to two-electron oxidation products^51,53^.

### Photochemical studies in solution and in solid state

**Figure 5:**
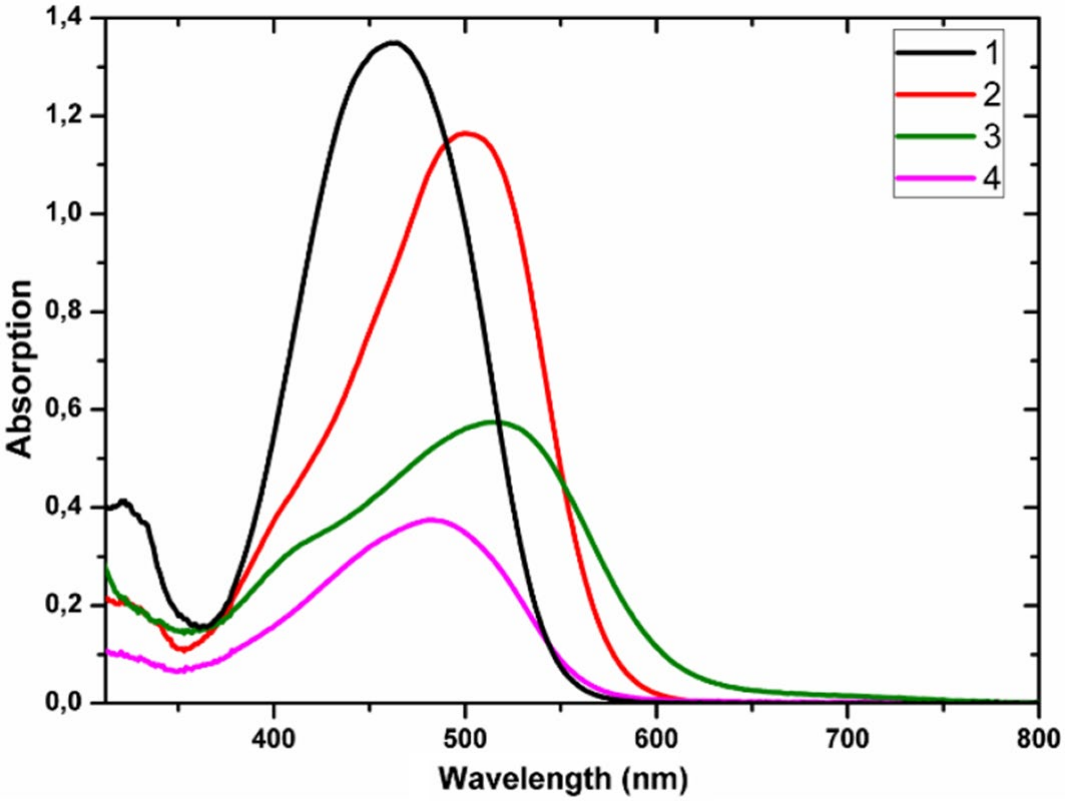
UV–visible absorption spectroscopy of compounds **1–4**.

The UV–vis absorption spectra of the azo-ethynyl complexes **1–4** were recorded in dichloromethane, and the absorption data of these compounds are listed in Table 2. The ferrocenyl compound **1** (Fig. 5) shows a wide and strong absorption band between 390–530 nm, presumably due to superimposed π–π* and d–d transition arising from the ferrocene moiety^54–57^, together with the n–π* and π–π* absorption bands of the azobenzene chromophore.

**Table 2:** The values of *χ*^*(2)*^_*eff*_, *χ*^*(3)*^_*elec*_*,* and *γ*_*elec*_ obtained for **1**, **2**, **3** and **4** guest–host films.

Compounds	*χ*^*(2)*^_*eff*_, *s–p *(pm V^−1^)	*χ*^*(2)*^_*eff*_, *p–p *(pm V^1^)	*χ*^*(3)*^_*elec*_, *s–s *(10^–20^ m^2^V^−2^)	*γ*_*elec*_ (10^–47^ *m*^5^V^−2^)
**1**	0.15	1.46	0.61	5.55
**2**	0.09	0.69	0.35	3.22
**3**	0.05	0.35	0.25	2.26
**4**	0.99	5.41	0.27	2.44

The UV–Vis spectra for the acetylide derivatives **1–4** are summarized in Table 1 and presented in Fig. 5. They are very similar with those of parent compounds mentioned in the literature.

The UV–vis spectrum of complex **2** contains a broad low energy non-symmetrical absorption band with a maximum centred around 514 nm. This low energy band is accompanied by a smooth shoulder at 400 to 430 nm with can be consistent with the π–π* absorption bands of the ethynyl-azo unit as observed in *trans*-[RuCl(-C⋮C-R)(dppe)_2_]^14^, that overlaps with MLCT bands of the metal-acetylide unit.

Like the parent *cis*-[RuCl_2_(PPh_3_)_2_(4,4′-dimethyl-2,2′-bipyridine)] complex^34,51^, **3** has a major absorption band in the 450 − 550 nm range (λmax = 514 nm), that can been attributed to ruthenium-to-bipyridyl MLCT transitions^51^. This broad band is accompanied by a distinct shoulder with an apparent maximum at 400 nm which can be consistent with the absorption bands of the azobenzene chromophore.

The UV–vis spectra for the alkynyl-metal complex **4** shows a broad absorption band from 380 to 550 nm, that would correspond to the superimposition of both a MLCT band around 400 nm, that is concordant with the data mentioned for other Ru(-C⋮C-R) (PPh_3_)_2_(*η*^*5*^-C_5_H_5_) (R = H, CHO) complexes^52^, and the π–π* absorption bands of the azobenzene chromophore, as observed in the previous compounds.

**Figure 6:**
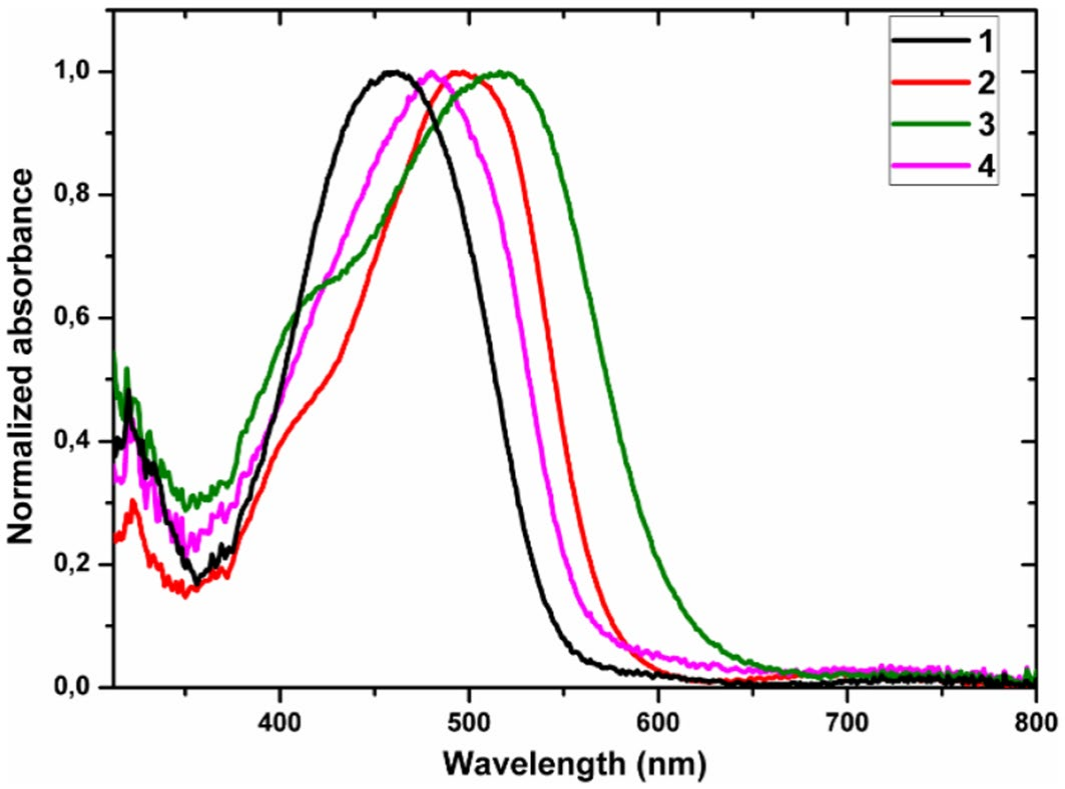
Normalized UV–vis spectra of the compounds **1**, **2**, **3** and **4** incorporated in PMMA films at the concentration 100 µmol/g.

The UV–Visible absorption spectra of the four investigated metal complexes dissolved in dichloromethane and then embedded in PMMA films at the concentration 100 µmol/g are given in Fig. 6. The spectra of the obtained films exhibit wide absorption band with maximum at 464 nm, 500 nm, 514 nm, and 481 nm respectively for the films of complexes **1**, **2**, **3** and **4** which correspond to ligand centered (LC, π–π* and n–π*) transitions^58^. They are absolutely similar to the absorption spectra obtained for the four investigated metal complexes dissolved in dichloromethane. More interestingly for the measurements of SHG and THG, we observed that the samples show high optical transparency, at the wavelengths higher than 700 nm.

### SHG and THG responses

**Figure 7:**
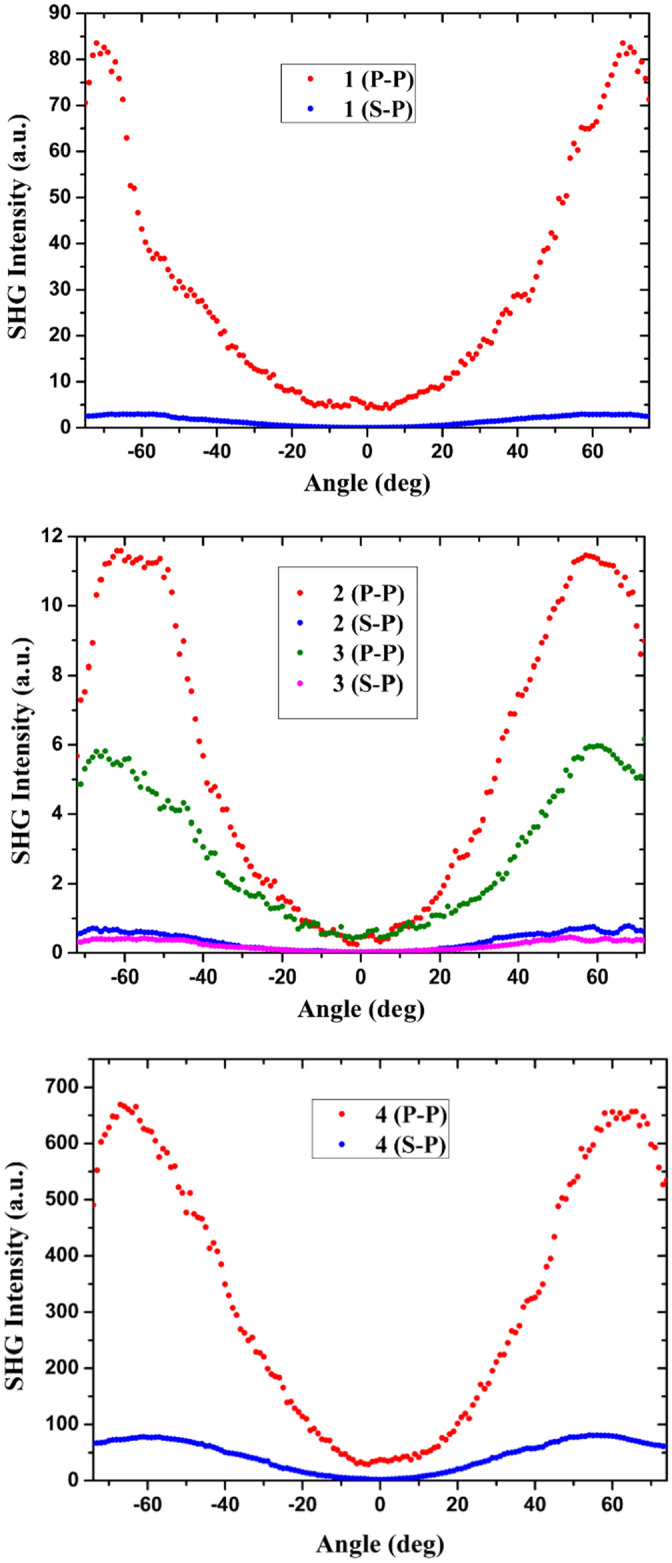
SHG intensity as a function of incident angle in **1**, **2**, **3** and **4** guest–host films at the *s*-, and *p*-polarized fundamental beam.

The measurements of SHG and THG of compounds **1**, **2**, **3** and **4** in PMMA films at the concentration 100 µmol/g, were obtained by using the Maker fringe technique for s- and p-polarized fundamental beam. We used the corona poling just before starting the measurements in order to increase a uni-axial orientation of the molecules and consequently enhance the 2nd order NLO properties of the compounds in the polymer films. The obtained SHG response is due to the break of the centrosymmetry of the studied organometallic complexes. Figure 7 represents the dependence of the second harmonic intensity generated as a function of the incident angle. As shown in this figure, the four films have a maximum signal between 60° and 65° and zero intensity at normal incidence of the fundamental beam. The intensity depends on the polarization p or s. In our case, the polarization of the second harmonic signal was found to be always p-polarized regardless the incident polarization.

For the calculation of the quadratic NLO susceptibilities, we used the comparative model of Lee and coworkers which compares the intensities of SHG for films and for quartz plate which is expressed by the following equation and which takes into account the linear optical absorption^59^.

1 $$\chi ^{{(2)}} = \chi _{{Quartz}}^{{(2)}} \left( {\frac{2}{\pi }} \right)\left( {\frac{{l_{{Quartz}}^{{coh}} }}{d}} \right)\left( {\frac{{\frac{{\alpha d}}{2}}}{{1 - e^{{ - \frac{{\alpha d}}{2}}} }}} \right)\left( {\frac{{I^{{2\omega }} }}{{I_{{Quartz}}^{{2\omega }} }}} \right)^{{1/2}}$$

where ${\chi }_{Quartz}^{(2)}=1.0 \text{pm/v}$ is the quadratic susceptibility of quartz, ${l}_{Quartz}^{coh}$ is the coherent length of quartz, *α* is the absorption coefficient at doubled laser wavelength, *d* is the film thickness, ${I}^{2\omega }$ and ${I}_{Quartz}^{2\omega }$ are the SHG intensities for the sample and quartz respectively under the same conditions of measurement. The obtained quadratic NLO susceptibilities for **1–4** films are presented in Table 2. For the input–output values of ${\chi }^{(2)}$ were found to be higher at polarization p-p than polarization s-p, which is caused by the symmetry peculiarities of the guest–host polymeric films after poling. On the basis of these results (Table 2) the film of complex **4** was found to exhibit higher second-order optical nonlinearity than the three other films. This result might be due to different polarity and hence different charge transfer from the metal to the π-conjugated acetylide azobenzene system (MLCT) in complex **4** as compared with the other two ruthenium metal complexes. This is intrinsic to the presence of bipyridine (**3**) or cyclopentadienyl (**4**) pi-acceptor ligands, whereas the dppm (**2**) and PPh_3_ ligands are sigma-donor ligands. The structure of **1** is different since there is no metal–carbon bond between iron and acetylide. The charge transfer will be weak there, as the dibutylamino moiety acts as a strong electron donor unit. In fact in complex **3** the CV and the UV–visible measurements indicate a charge transfer from ruthenium to the bipyridyl unit^50^.

**Figure 8:**
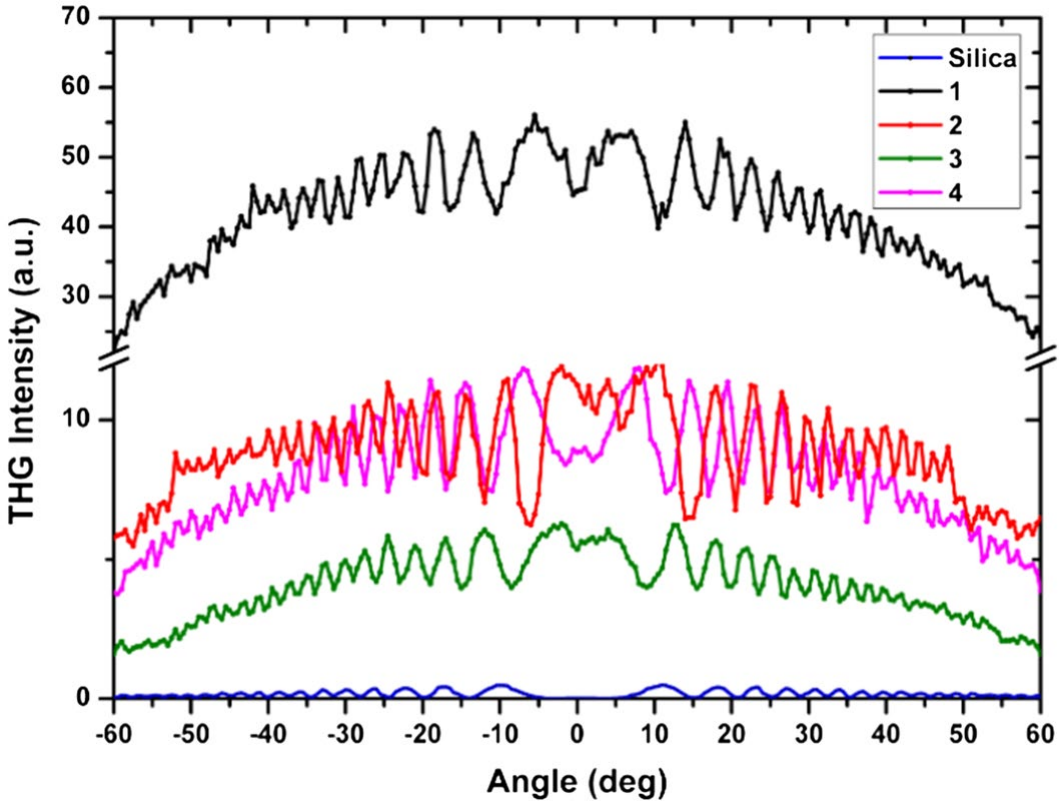
THG intensity as a function of incident angle in **1**, **2**, **3** and **4** guest–host films.

For the calculation of cubic NLO susceptibilities, we used the model of Kubodera and Kobayashi. This model compares directly the maximum amplitudes of third harmonic light intensities of the studied material with those of flat silica which is taken a reference (1 mm in thickness). This model takes into account the optical absorption of the studied compounds^60^:

2 $$\chi ^{{(3)}} = \chi _{{Silica}}^{{(3)}} \left( {\frac{2}{\pi }} \right)\left( {\frac{{l_{{Silica}}^{{coh}} }}{d}} \right)\left( {\frac{{\frac{{\alpha d}}{2}}}{{1 - e^{{ - \frac{{\alpha d}}{2}}} }}} \right)\left( {\frac{{I^{{3\omega }} }}{{I_{{Silica}}^{{3\omega }} }}} \right)^{{1/2}}$$

where ${\chi }_{Silica}^{(3)}$ is the cubic susceptibility of silica, ${l}_{Silica}^{coh}$ is the coherent length of silica, *α* is the absorption coefficient at triple laser wavelength, *d* is the film thickness, ${I}^{3\omega }$ and ${I}_{Silica}^{3\omega }$ are the THG intensities of the samples and silica at the same conditions, respectively. The Fig. 8 represents the dependences of the third harmonic intensity generated as function of the incident angle.

The calculated values of ${\chi }^{(3)}$ for **1–4** films are given in Table 2. We have the same results before and after corona poling which is expected as the orientation has no significant influence on third-order NLO properties. Due to the extended $\pi$-conjugated chain complexes and coupling between the metal^61^ and the remote groups through the $\uppi$ -conjugated path of the investigated metal complexes, the third-order nonlinear optical susceptibilities values of ${\chi }^{(3)}$(Table 2) are stronger than the one of the silica^62^. The complex **1** is characterized with a higher third order NLO susceptibility than **2**, **3** and **4** due to more effective electronic delocalization which is caused by charge transfer from the (N,N-dibutylamine) and ferrocenyl donor groups to the azo accepting unit. from third order susceptibility (Table 2) and taking into account the concentration of the complexes and the local field factor, we calculated the electronic contribution of second hyperpolarizability *γ*_*elec*_ and we found that third order susceptibility, *γ*_*elec*_ for **1** is also much higher than the other complexes^62,63^.

Although the experimental methods used in the literature to determine the ${\upchi }^{(2)}$ and ${\upchi }^{(3)}$ susceptibilities are slightly different, a comparison between the performances of our four organometallic complexes **1–4** and the ones reported for the state-of-the-art SHG/THG compounds could be made. However we have to underline here that for the third harmonic generation as we investigate thin film so what we are measuring is electronic contributions which is not the case for example for degenerate four waves mixing but comparison could be done. From Table 3, we can clearly see that the ${\upchi }^{(3)}$ susceptibility values are slightly lower in the case of our organometallic complexes while the ${\upchi }^{(2)}$ susceptibility values are generally much higher for our complexes **1–4**. Thus, the sizeable SHG response for the complexes **1** and **4** indicates that these compounds are valuable candidates for potential applications in NLO and more specifically, for the evolution towards applications in molecular electronics and photonics and in related fields including second-order optical effects in organometallic compounds induced by an acoustic field^26,64^. Considering third harmonic generation, electronic contributions were measured as we investigated thin films. This is obviously a difference with results previously reported by using the degenerate four waves mixing approach^65–71^.

**Table 3:** Comparison of the SHG/THG NLO properties of **1–4** compounds with the values of some selected compound in the literature (AISHG: acoustically induced second harmonic generation/DFWM: degenerate four wave mixing/AIOSHG: acoustically induced optical second harmonic generation/EFISH: electric field induced second harmonic).

Compounds	$\uplambda (\mathrm{nm})$	Technique	${\chi }^{(2)}$ (pm V^−1^)	${\chi }^{(3)}$ (10^–20^ m^2^ V^−2^)
**1**	1,064	SHG/THG	1.46	0.61
**2**	1,064	SHG/THG	0.69	0.35
**3**	1,064	SHG/THG	0.35	0.25
**4**	1,064	SHG/THG	5.41	0.27
Trans Ru(2,5-C≡CC-th-CHO)Cl(dppe)_2_^26^	1,760/532	AISHG	0.4	–
Trans Ru(2,2′,5,5′-C≡C-th-th-CHO)Cl(dppe)_2_^12, 21,26^	1,760/532	AISHG/DFWM	0.8	1.2
Trans Ru(2,2′,5,5′,2′′,5′′-C≡C-th-th-th-CHO)Cl(dppe)_2_^26^	1,760/532	AISHG/DFWM	0.27	2.10
Trans Ru(-C≡C-th-(E)CH = CH-th-CHO)Cl(dppe)_2_^26^	1,760/532	AISHG/DFWM	0.64	2.50
C_30_H_28_N_4_O_4_Sn^72^	1,300	THG	–	1.99
Pb_3_Ge_5_O_12_^26^	1,760	AIOSHG	0.61	–
Ru − X(CuCR)(dppe)_2_^21^	1,064	EFISH	1.4	–
LiNbO_3_^26^	1,760	AIOSHG	0.12	–

### Hyperpolarizabilities and HOMO, LUMO analysis

To engineer optimal nonlinear optical properties, the origins of the nonlinear optical phenomena must be understood. This requires knowledge of the bonding properties of the atoms in the molecules. To understand the connection between molecular structure and NLO property, we have to expand the scope of our investigation to include the computing the first and second order hyperpolarizabilities (β, γ). The obtained theoretical results are collected in Tables 4, 5. The *β*_*tot*_ value has been calculated using the following expression^73^.

**Table 4:** Some selected components of the frequency-dependent β (− 2ω;ω,ω) values at ω = 0.042827 atomic unit (1,064 nm) for compounds **1–4**.

Compounds	β_x_ × 10^–30^ esu	β_y_ × 10^–30^ esu	β_z_ × 10^–30^ esu	β_tot_ × 10^–30^ esu
**1**	0.803	− 2.759	− 212.401	212.420
**2**	− 3.786	− 1.414	− 66.023	66.146
**3**	51.038	1.3107	− 18.692	54.369
**4**	70.634	− 1.444	− 58.023	91.422
Trans[Ru(4-CCC6H4CHO)Cl(dppe)_2_				40^74^
Trans[Ru(4-CCC6H_4_NO_2_)Cl(dppe)_2_				55^74^
Organometallic complexes				10–1,300^74^ 5.7–140^30^

**Table 5:** Some selected components of the frequency-dependent γ (−2ω;ω,ω,0) values at ω = 0.042827 atomic unit (1,064 nm) for compounds **1–4.**

Compouds	γ_xxxx_ × 10^–36^ esu	γ_yyyy_ × 10^–36^ esu	γ_zzzz_ × 10^–36^ esu	γ_xxyy_ × 10^–36^ esu	γ_xxzz_ × 10^–36^ esu	γ_yyzz_ × 10^–36^ esu	γ_tot_ × 10^–36^ esu
**1**	4.59	6.32	1,600.73	1.18	− 11.54	8.57	321.59
**2**	52.59	49.03	1507.85	9.73	14.74	15.11	337.73
**3**	951.55	61.87	106.64	5.17	279.13	3.69	339.21
**4**	1,338.12	31.63	24.50	17.23	46.12	8.48	307.59
Trans[Ru(4-CCC6H4CHO)Cl(dppe)_2_							300 ^75^
Trans[Ru(4-CCC6H_4_NO_2_)Cl(dppe)_2_							320 ^75^
Organometallic complexes							9–360 ^65^ 5.5–100 ^30^

3 $${\beta }_{tot}=\sqrt{{\beta }_{x}^{2}+{\beta }_{y}^{2}+{\beta }_{z}^{2}}$$

Generally, an increase in the *β* value occurs together with the hypsochromic shift effect (see Table 4) what is seen for compounds **1** and **4**. Theoretical results suggest that better second order hyperpolarizabillities possess the ferrocenyl compound **1** and the ruthenium metal complex **4**. These results are in good agreement with the experimental results, where the best *χ*^*(2)*^ were obtained for molecules **1** and **4**. The HOMO (Highest Occupied Molecular Orbital) and LUMO (Lowest Unoccupied Molecular Orbital) are very important aspects to consider the NLO properties. In general if the HOMO–LUMO gap decreases (molecule **2–3**), thus causing a bathochromic shift the NLO properties should increase. In our case, there is no such relationship because absorption bands for molecules **2**, **3** are close to resonance and the second harmonic signal can be partially absorbed.

The calculated hyperpolarizabilities of these molecules were compared with first order hyperpolarizabilities of other organometallic compounds (see Table 4 ^30,74^). The study reveals that the investigated complexes have the same *β* range as other organometallics complexes known in literature hence in general may have potential applications in the development of non-linear optical materials.

The average second-order hyperpolarizability *γ*_*tot*_ values have been calculated using the following formula:

4 $${\gamma }_{tot}=\left(\frac{1}{5}\right)\left[{\gamma }_{xxxx}+{\gamma }_{yyyy}+{\gamma }_{zzzz}+2\left({\gamma }_{xxyy}+{\gamma }_{xxzz}+{\gamma }_{yyzz}\right)\right]$$

The calculated second order hyperpolarizability tensor components are presented in Table 5. The hyperpolarizability of γ_tot_ obtained for molecules **1–4** are quite large comparable to the values obtained for other organometallic materials as presented in Table 5 ^30,61^. The second order frequency-dependent hyperpolarizability for compound **1** and **2** is dominated by the longitudinal component of γ_zzzz_, but for the molecule **3** and **4** the maximal tensor component is achieved for γ_xxxx_. The same conclusions can be drawn in relation to the components obtained for the calculation of the first hyperpolarizability. Domination of particular component indicates a substantial delocalization of charges in these directions. This electron distribution can be skewed by substituents, the enlarge of this redistribution is measured by dipole moment, and facility of redistribution in response to an externally applied electric field by hyperpolarizability.

**Figure 9:**
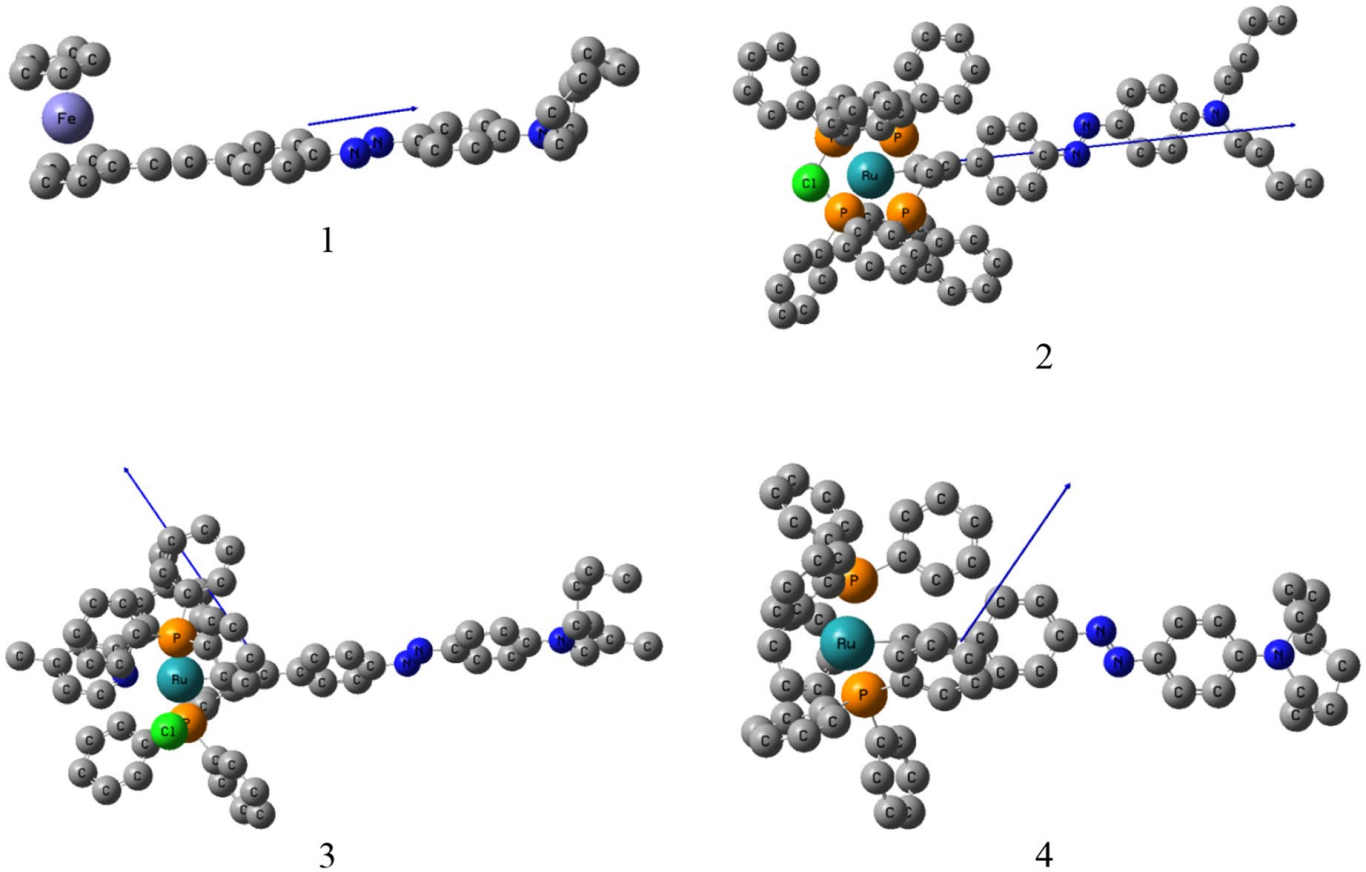
The electric dipole moment vector of **1–4**.

From Fig. 9 we can see the direction of the electric dipole moment. It can be deduced also from Table 6 that for the molecules **1** and **2**, the dipole moment vectors are directed mainly along the longest part of the molecule μ_z_ while for molecules **3**, **4**, the dipole moment vectors in the direction of phosphine ligands μ_x_ If we take into account the maximum values of γ_zzzz_ for **1** and **2** and γ_xxxx_ for **3**, **4** a good agreement is obtained between theoretical and experimental results.

**Table 6:** The calculated electric dipole moments (Debye) and dipole moment components for complexes 1–4. (DFT/B3LYP levels in 6-31G (d,p) + lanl2dz basis set).

Compounds	µ_x_ (Debye)	µ_y_ (Debye)	µ_z_ (Debye)	µ_tot_ (Debye)
**1**	0.15	0.65	− 4.48	4.53
**2**	0.03	− 0.33	5.21	5.21
**3**	9.70	0.44	5.10	10.96
**4**	4.90	− 3.50	− 1.53	6.42

HOMO, LUMO and HOMO–LUMO-energy gaps have been also calculated in order to analyze the relationship of NLO properties with the molecular structure of the studied compounds. Interpretations of orbital energies give useful forecasting. As usually, the HOMO and LUMO energy levels depend on electron donating strength of donor and electron – withdrawing strength of acceptor, respectively.

The energy gap (∆*E*_*L*−*H*_ = *E*_*LUMO*_ − *E*_*HOMO*_), explains the charge transfer interaction taking place within the ligands. The HOMO represents the ability to donate an electron and the LUMO represents the ability to obtain an electron. The HOMO and LUMO energies and HOMO–LUMO energy gaps are given in Table 6. The width of the energy gap obtained for investigated materials falls within limits 2–3 eV. Which correlates well with the maximum in the absorption spectrum, see Table 1. The reduction in the HOMO–LUMO energy gap explains eventually charge transfer interaction taking place within the molecules. Generally, if conjugation bonds in a molecule increases, the HOMO–LUMO gap decreases and leads to a bathochromic shift. The HOMO–LUMO energy gap decreases also upon metal complexation (see Table 7) E_LUMO_-E_HOMO_ for free ligand is higher than for complexes **1–4** what should be related to increasing of NLO properties.

**Table 7:** HOMO, LUMO energies (E_HOMO_, E_LUMO_) and HOMO–LUMO gaps (E_LUMO_-E_HOMO_) calculated at the DFT/B3LYP levels in 6-31G (d) and lanl2dz basis set.

Compound	E_HOMO_ (eV)	E_LUMO_ (eV)	E_LUMO_–E_HOMO_ (eV)
Ligand	− 7.24111	− 1.70347	5.53764
**1**	− 5.06633	− 2.16036	2.90597
**2**	− 4.48454	− 1.74456	2.73998
**3**	− 4.18738	− 2.13342	2.05396
**4**	− 5.519867	− 2.51445	3.00541

Based on the obtained experimental and theoretical results (see Supporting Information pages S10-S11) we can deduce that in our case the large second and third order optical nonlinearity is caused by strong intramolecular charge transfer between donors and acceptors through a conjugated transmitter.

The theoretical results show that the geometry of the molecule can determine nonlinear properties. The choice of the auxiliary ligands allows the direction of dipole moment of the complex to be modified and it can change the linear and nonlinear optical properties the whole system. Comparison of theoretical results with the values published in the literature indicates that all these complexes exhibit large microscopic and macroscopic second and third-order NLO properties.

## Conclusion

In summary, we have reported herein the synthesis and full characterization of four specific organometallic complexes (**1–4**). The UV–Visible absorption spectra as well as the cyclic voltammetry measurements indicated a different metal to ligand charge transfer (MLCT). Thus, the second and third-order nonlinear optical properties of these four organometallic complexes have been evaluated by means of second and third harmonic generation (SHG and THG) techniques. The values of the second order NLO susceptibility of our four complexes were found to be the highest for complex **4** while complex **1** showed the strongest third harmonic generation. The difference of obtained values of second and third nonlinear susceptibilities is due to modification of the energy states and the introduction of a charge transfer state upon complexation which are of different nature within the four metal complexes. Calculations correctly reproduce HOMO, LUMO energies and hyperpolarizabilities of the organometallic metal complexes **1–4**. The calculated HOMO and LUMO energies show that charge transfer occurs within the molecules. From theoretical calculation, it is clear that the presence of two Ph_3_P acting as the most electron-acceptor groups in **3** and **4** complexes skew the direction of the dipole moment causing electron delocalization in other direction than for the molecule **1** and **2**. The possible modulation of the nonlinear optical susceptibility by the nature of the metal atom used and its coordination sphere is an important phenomenon which opens new perspectives for both photonic and opto-electronic applications.

## Supplementary information

Supplementary Information.
