# Author Correction: Transition metals induce control of enhanced NLO properties of functionalized organometallic complexes under laser modulations

**DOI:** 10.1038/s41598-021-00997-x

**Published:** 2021-10-26

**Authors:** S. Taboukhat, N. Kichou, J.‑L. Fillaut, O. Alévêque, K. Waszkowska, A. Zawadzka, A. El‑Ghayoury, A. Migalska‑Zalas, B. Sahraoui

**Affiliations:** 1 MOLTECH-Anjou, UMR 6200, CNRS, Univ Angers, 2 Bd Lavoisier, 49045 Angers, France; 2 Univ Rennes, CNRS, ISCR-UMR 6226, 35000 Rennes, France; 3 Department of Chemistry, Faculty of Sciences, University of Mouloud Mammeri, 15000 Tizi‑ouzou, Algeria; 4 Institute of Physics, Faculty of Physics, Astronomy and Informatics, Nicolaus Copernicus University, 5 Grudziadzka, 87-100 Torun, Poland; 5 Faculty of Science and Technology, Jan Dlugosz University in Czestochowa, Al. Armii Krajowej 13/15, 42‑200 Czestochowa, Poland

Correction to: *Scientific Reports* 10.1038/s41598-020-71769-2, published online 17 September 2020

The original version of this Article contained an error in Affiliation 4, which was incorrectly given as ‘Department of Automatic and Measurement Systems, Faculty of Physics, Astronomy and Informatics, Nicolaus Copernicus University, Grudziadzka 5, 87-100, Torun, Poland’. The correct affiliation is listed below:

Institute of Physics, Faculty of Physics, Astronomy and Informatics, Nicolaus Copernicus University, 5 Grudziadzka, 87-100 Torun, Poland.

The original Article has been corrected.

