# Supplementary material for: Transition metals induce control of enhanced NLO properties of functionalized organometallic complexes under laser modulations
Source: Sci Rep. 2020 Sep 17;10:15292. doi: 10.1038/s41598-020-71769-2 (PMC7499250; doi:10.1038/s41598-020-71769-2)
Supplement: Supplementary file 1 — Supplementary Information. [file 41598_2020_71769_MOESM1_ESM.doc]

**Transition Metals Induce Control of Enhanced NLO Properties of Functionalized Organometallic Complexes under Laser modulations**

***S. Taboukhat, N. Kichou, J.-L. Fillaut, O. Alévêque, K. Waszkowska, A. Zawadzka, A. El-Ghayoury, A. Migalska-Zalas and B. Sahraoui***

A Table of Content

.


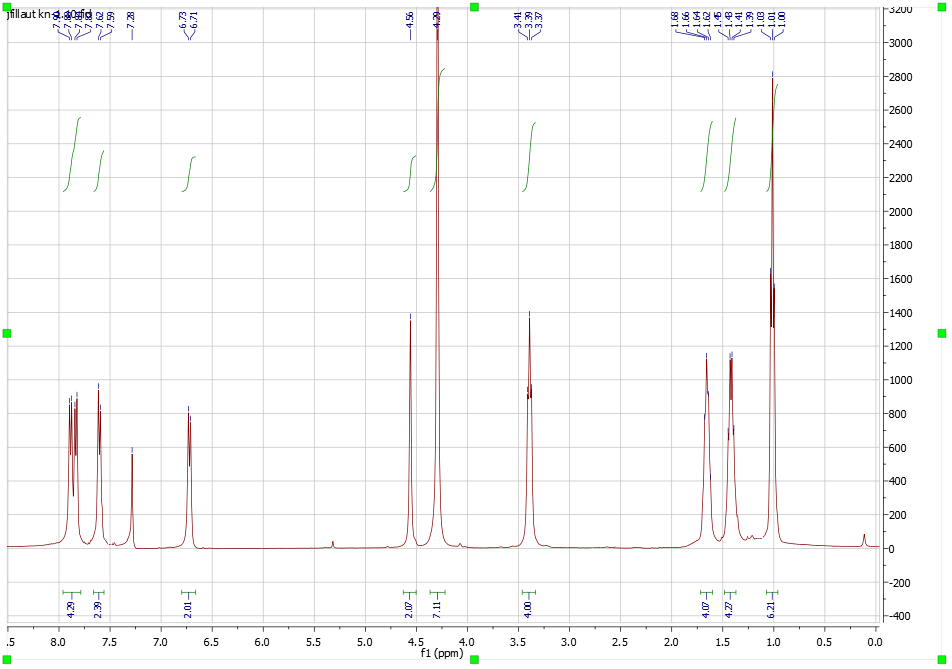


^1^H NMR (400 MHz, Chloroform-*d*) δ 7.89 (d, 7.8Hz, 2H), 7.84 (d, 7.8Hz, 2H), 7.62 (d, 8.0 Hz, 2H), 6.72, (d, 8.0 Hz, 2H), 4.56 (s, 2H), 4.29 (br. s., 7H), 3.39 (t, 7.8 Hz, 4H) , 1.66 (m, 4H) 1.43 (m, 4H), 1.01 (t, 7.7 Hz, 6H).

Figure 1: ^1^H NMR of complex **1.**


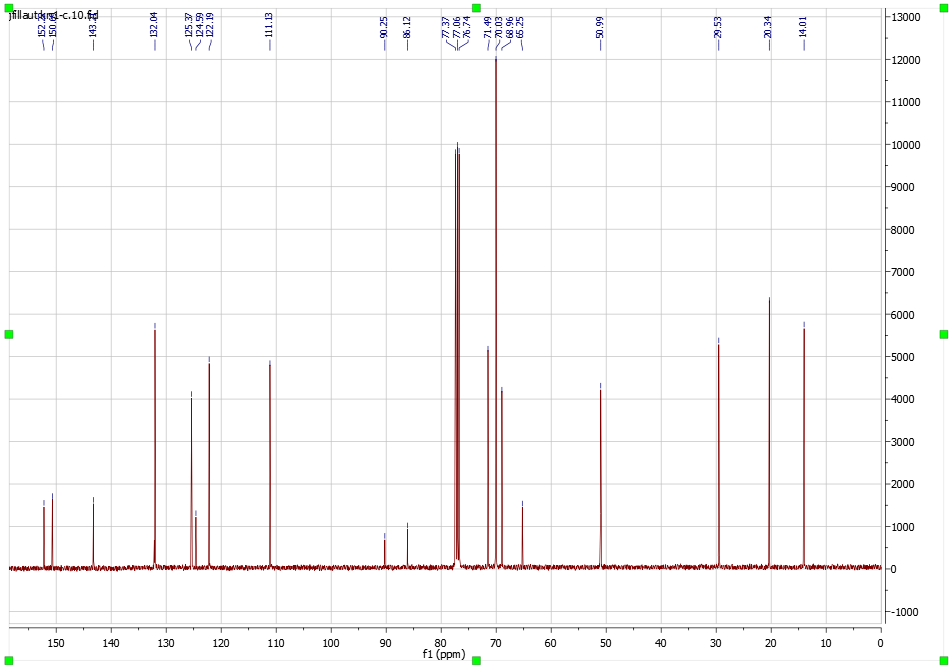


^13^C NMR (101 MHz, Chloroform-*d*) δ 152.22, 150.69, 143.21, 132.04, 125.37, 124.59, 122.19, 111.13, 90.25, 86.12, 71.49, 70.03, 68.96, 65.25, 50.99, 29.53, 20.34, 14.01.

Figure 2: ^13^C NMR of complex **1.**


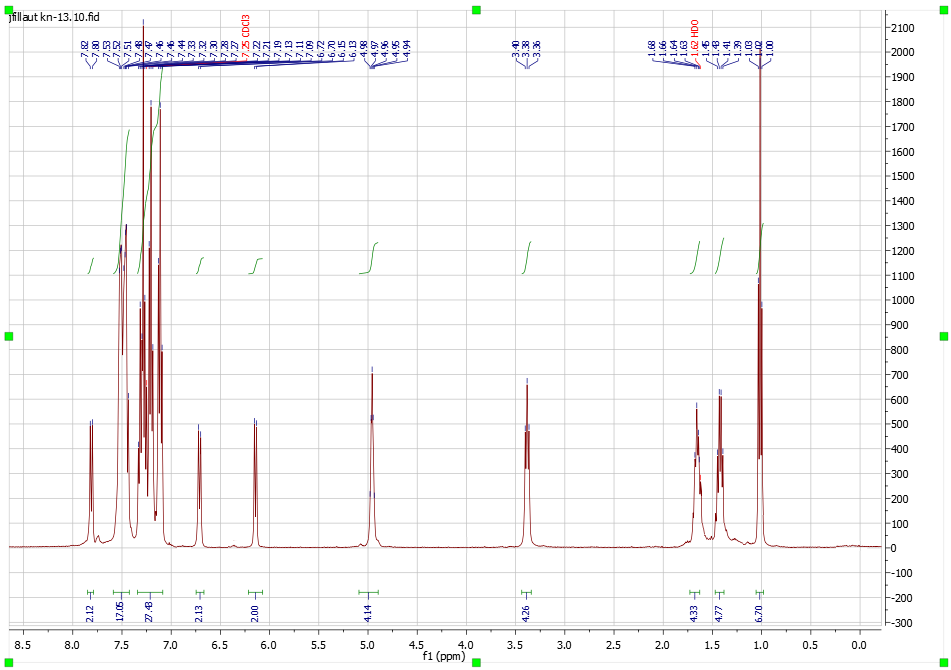


^1^H NMR (400 MHz, Chloroform-*d*) δ 7.81 (d, 7.8 Hz, 2H), 7.53-7.44 (m, 16H) 7.32 (t, 8.0 Hz, 4H), 7.30 (t, 8.0 Hz, 4H), 7.28 (t, 8.0 Hz, 8H), 7.21(t, 8.0 Hz, 8H), 7.11 (t, 7.2 Hz, 2H), 6.72 (d, 7.8 Hz, 2H), 6.14 (d, 7.8 Hz, 2H), 4.96 (m., 4H), 3.38 (d, 7.8 Hz, 2H), 1.66 (m., 4H) 1.42 (m., 4H), 1.02 (t., 7.0 Hz, 6H).

^31^P NMR (162 MHz, Chloroform-*d*) δ, -6.7 ppm

Figure 3: ^1^H NMR of complex **2.**


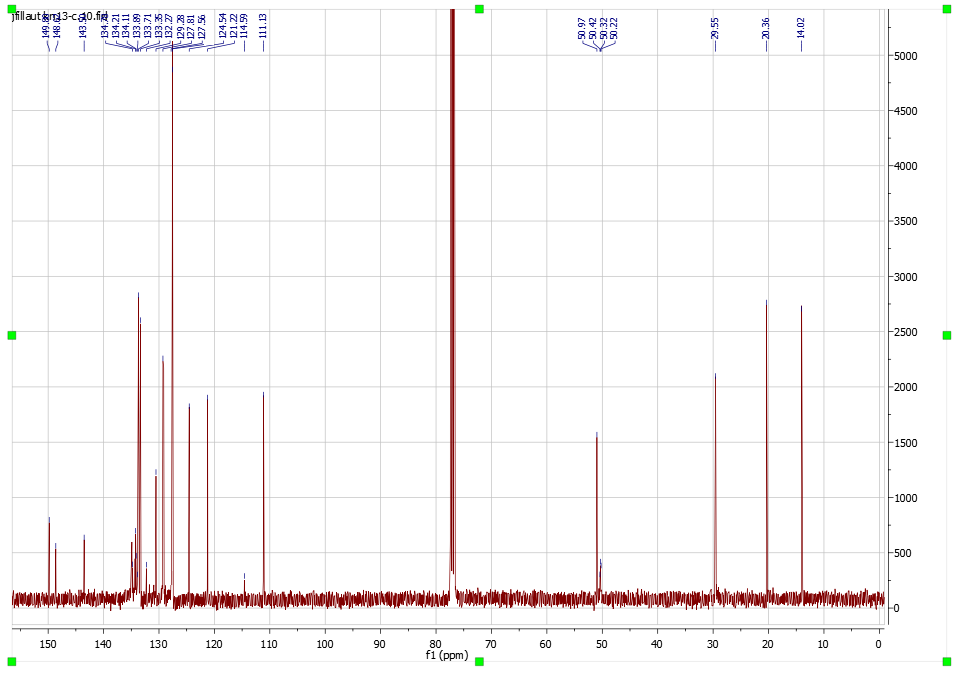


^13^C NMR (101 MHz, Chloroform-*d*) δ 149.8, 148.6, 143.5, 134.2 (quint , J_CP_ = 11Hz), 133.8 (quint., J_CP_ = 11Hz), 133.4, 132.3, 129.28, 127.81, 127.56, 124.54, 121.22, 114.59, 111.13, 50.97, 50.32 (t., J_CP_ = 10Hz), 29.55, 20.36, 14.02.

Figure 4: ^13^C NMR of complex **2.**


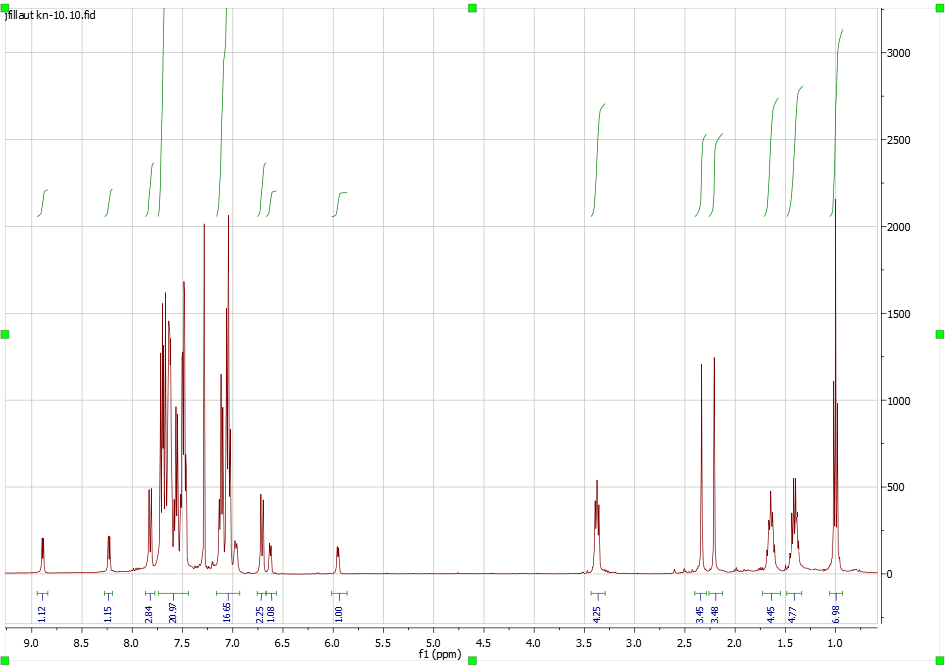
1H NMR (400 MHz, Chloroform-*d*) δ 8.90 (d, 5.5 Hz, 1H), 8.22 (d, 5.5 Hz, 1H), 7.81 (d, 7.8 Hz, 2H), 7.72-7.46 (unresolved m., 20H), 7.16 – 6.93 (unresolved m.,16H), 6.72 (d, 7.8 Hz, 2H), 6.63 (d, 5.5 Hz, 1H), 5.95 (d, 5.5 Hz, 1H), 3.38 (t, 7.2 Hz, 4H), 2.33 (s, 3H), 2.21 (s, 3H), 1.65 (s, 4H), 1.42 (s, 4H), 1.00 (s, 7.8 Hz, 6H).

^31^P NMR (162 MHz, Chloroform-*d*) δ, 29.5 ppm.

Figure 5: ^1^H NMR of complex **3.**

13C
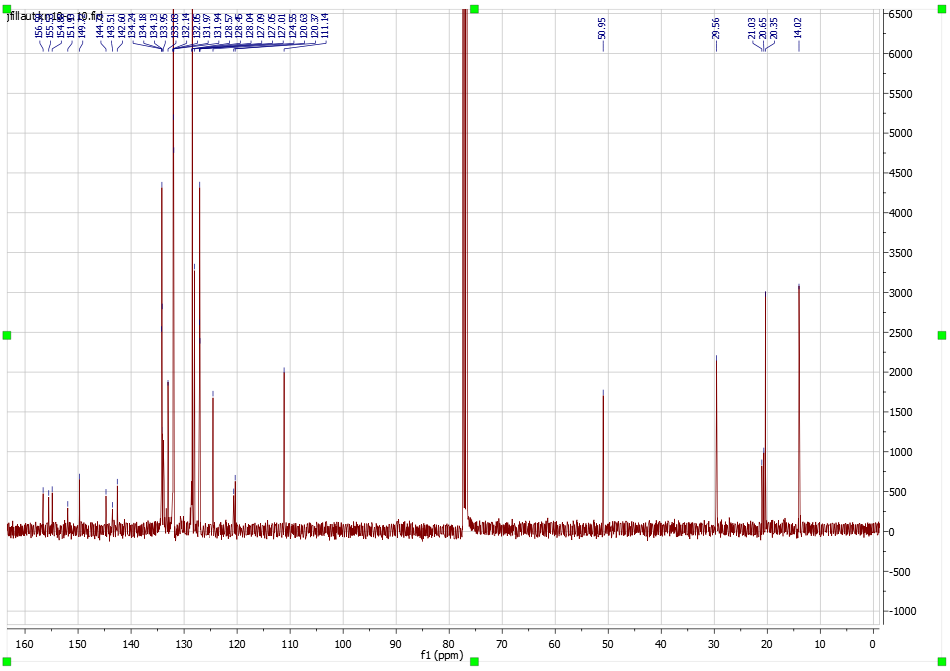


^13^C NMR (101 MHz, Chloroform-*d*) δ 156.59, 155.53, 154.88, 151.93, 149.72, 144.73, 143.51, 142.60, 134.18 (t, 4.2 Hz), 133.95, 133.03, 132.14, 132.05, 131.97, 131.94, 128.57, 128.45, 128.04, 127.05 (t, 4.0 Hz), 124.55, 120.63, 120.37, 111.14, 50.95, 29.56, 21.03, 20.65, 20.35, 14.02.

Figure 6: ^13^C NMR of complex **3**


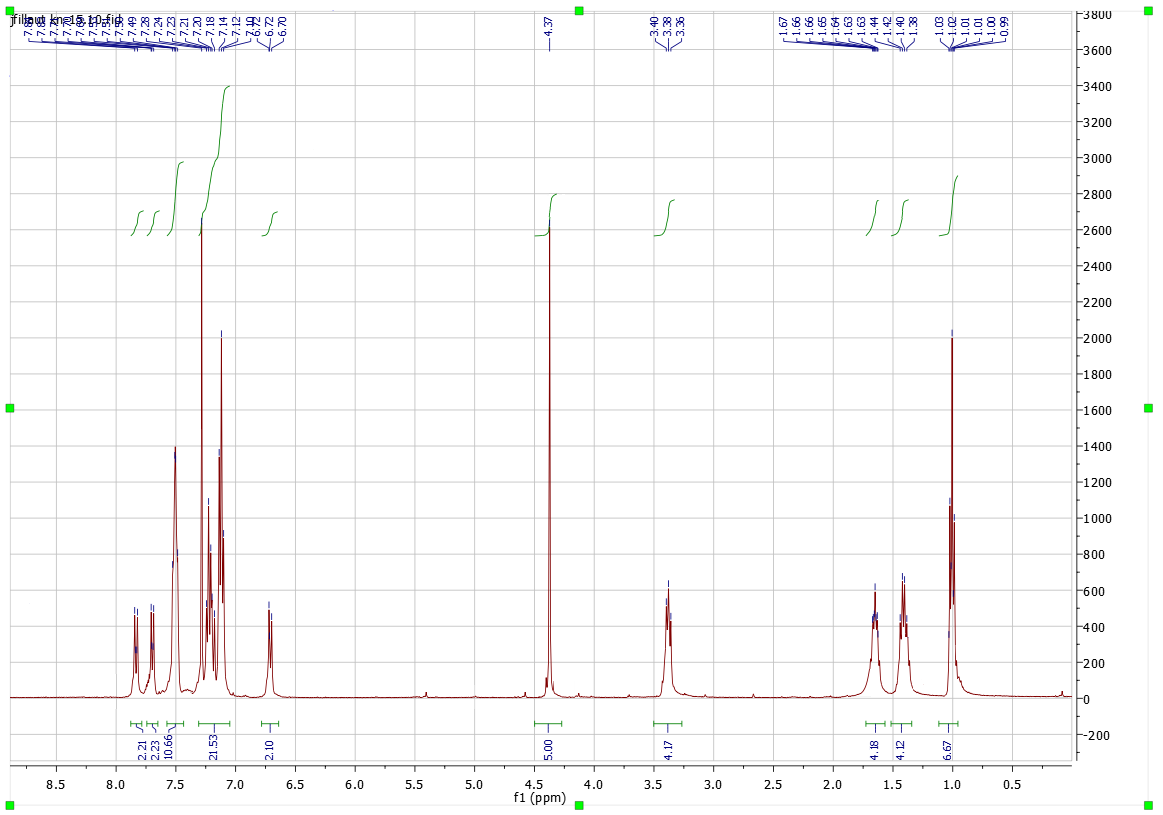
1H NMR (400 MHz, Chloroform-*d*) δ 7.83 (d, 7.8Hz, 2H), 7.70 (d., 7.8Hz, 2H), 7.51 (m, 10H ), 7.23 (m, 8H ), 7.16 (m, 12H), 6.71 (d, 7.8Hz, 2H), 4.37 (s, 5H) 3.38 (t, 7.0 Hz, 4H), 1.66 (m, 4H), 1.42 (m, 4H), 1.01 (t, 7.1 Hz, 6H).

^31^P NMR (162 MHz, Chloroform-*d*) δ, 50.2 ppm

Figure 7: ^1^H NMR of complex **4.**


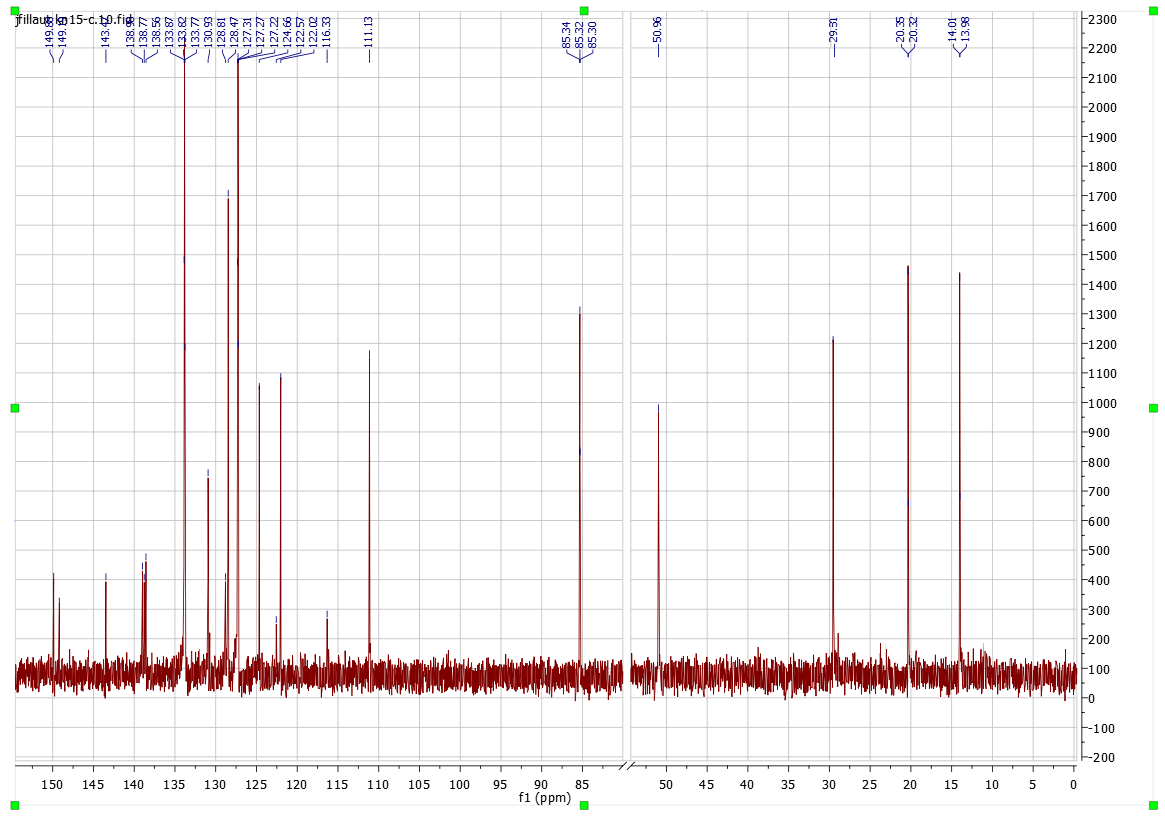


^13^C NMR (101 MHz, Chloroform-*d*) δ 149.88, 149.19, 143.47, 138.77 ( m, ^1^*J*_CP_ + *^3^J*_CP_ = 42 Hz), 133.82, 130.93, 128.81, 128.47, 127.27, 124.66, 122.57, 122.02, 116.33, 111.13, 85.32, 50.96, 29.51, 20.35, 14.01.

Figure 8: ^13^C NMR of complex**4.**

| Compounds | HOMO | LUMO |
| --- | --- | --- |
| **1** | 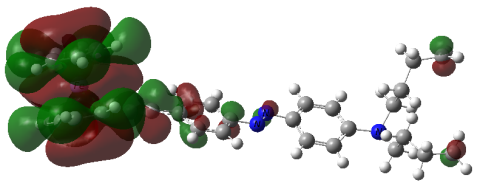 | 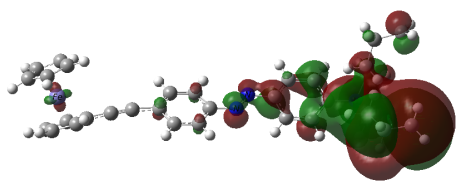 |
| **2** | 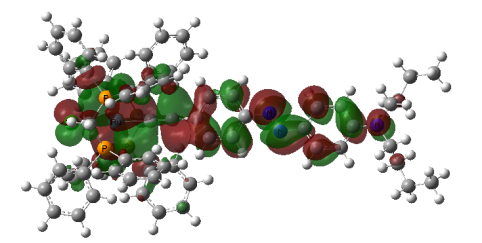 | 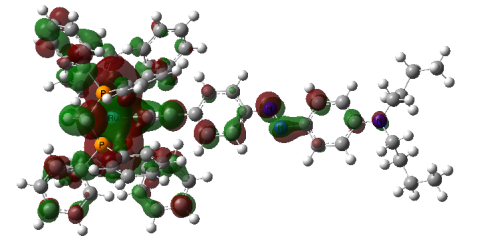 |
| **3** | 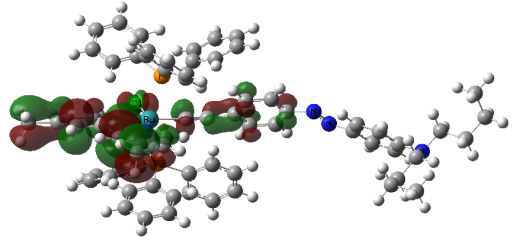 | 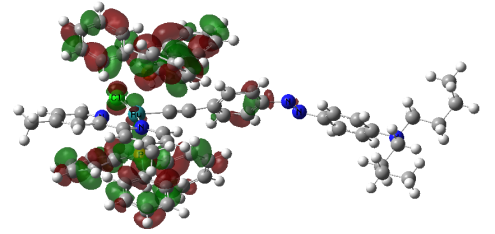 |
| **4** | 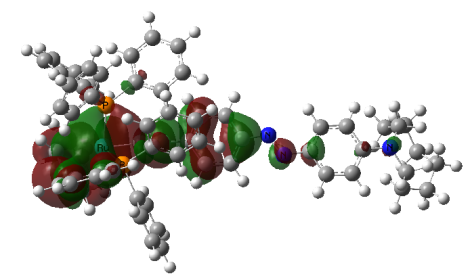 | 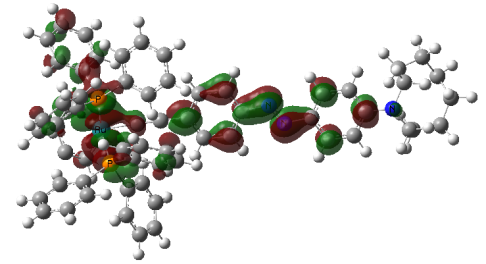 |

Figure 9: The frontier molecular orbitals: HOMO and LUMO for **1-4** compounds

The molecular design strategy is based on structural changes, such as manipulating the position of the electron donor and acceptor group and its ability to effectively intermolecular charge transport (ICT). Octahedral complexes studied in this work, have both σ-donor and π-accepting orbitals. This means that in such systems is possible both types of charge transfer transitions what causes the problem to recognize between LMCT (ligand to metal charge transfer) and MLCT (metal to ligand charge transfer). Our calculations are supported by the HOMO-LUMO diagram (see Table 7), where the maximum overlap of electron density between HOMO and LUMO energy levels is observed. From the contour plot (Table 7), it can be seen that the charge is significantly located on the ferrocenyl (molecule **1**) and is completely transferred to the (N,N-dibutylamine) group indicated on the LUMO plot. HOMO for compounds **2**, **3**, **4** comprises of the ruthenium organometallic part and azobenzene group for compound **2**. LUMO comprises of PPh2 or PPh3 ligands and of azobenzene fragment of compound **4** which pulls the charge from the ruthenium organometallic core. Hence it asserts that intramolecular charge transfer for compounds **1** and **4** is much significant among the ruthenium organometallic core and the azobenzene ligand. This type of charge transfer is desirable for second-order optical properties. For compound **2** we can notice the intramolecular charge transfer from azobenzene ligand to organometallic core (LMCT), while in both compound **2** and **3** the charge transfer towards the PPh2 (compound **2**) and PPh3 (compound **3**) groups is observed.
